# Impaired Antisaccades in Obsessive-Compulsive Disorder: Evidence From Meta-Analysis and a Large Empirical Study

**DOI:** 10.3389/fpsyt.2018.00284

**Published:** 2018-06-29

**Authors:** Katharina Bey, Leonhard Lennertz, Rosa Grützmann, Stephan Heinzel, Christian Kaufmann, Julia Klawohn, Anja Riesel, Inga Meyhöfer, Ulrich Ettinger, Norbert Kathmann, Michael Wagner

**Affiliations:** ^1^Department of Psychiatry and Psychotherapy, University of Bonn, Bonn, Germany; ^2^German Center for Neurodegenerative Diseases, Bonn, Germany; ^3^Department of Psychology, Humboldt-Universität zu Berlin, Berlin, Germany; ^4^Clinical Psychology and Psychotherapy, Freie Universität Berlin, Berlin, Germany; ^5^Biomedical Sciences and Psychology, Florida State University, Tallahassee, FL, United States; ^6^Department of Psychology, University of Bonn, Bonn, Germany; ^7^Department for Neurodegenerative Diseases and Geriatric Psychiatry, University Hospital Bonn, Bonn, Germany

**Keywords:** obsessive-compulsive disorder, OCD, antisaccade, endophenotype, meta-analysis, eye-tracking

## Abstract

Increasing evidence indicates that patients with obsessive-compulsive disorder (OCD) exhibit alterations in fronto-striatal circuitry. Performance deficits in the antisaccade task would support this model, but results from previous small-scale studies have been inconclusive as either increased error rates, prolonged antisaccade latencies, both or neither have been reported in OCD patients. In order to address this issue, we investigated antisaccade performance in a large sample of OCD patients (*n* = 169) and matched control subjects (*n* = 183). As impaired antisaccade performance constitutes a potential endophenotype of OCD, unaffected first-degree relatives of OCD patients (*n* = 100) were assessed, as well. Furthermore, we conducted a quantitative meta-analysis to integrate our data with previous findings. In the empirical study, OCD patients exhibited significantly increased antisaccade latencies, intra-subject variability (ISV) of antisaccade latencies, and antisaccade error rates. The latter effect was driven by errors with express latency (80–130 ms), as patients did not differ significantly from controls with regards to regular errors (>130 ms). Notably, unaffected relatives of OCD patients showed elevated antisaccade express error rates and increased ISV of antisaccade latencies, as well. Antisaccade performance was not associated with state anxiety within groups. Among relatives, however, we observed a significant correlation between antisaccade error rate and harm avoidance. Medication status of OCD patients, symptom severity, depressive comorbidity, comorbid anxiety disorders and OCD symptom dimensions did not significantly affect antisaccade performance. Meta-analysis of 10 previous and the present empirical study yielded a medium-sized effect (*SMD* = 0.48, *p* < 0.001) for higher error rates in OCD patients, while the effect for latencies did not reach significance owing to strong heterogeneity (*SMD* = 0.51, *p* = 0.069). Our results support the assumption of impaired antisaccade performance in OCD, although effects sizes were only moderately large. Furthermore, we provide the first evidence that increased antisaccade express error rates and ISV of antisaccade latencies may constitute endophenotypes of OCD. Findings regarding these more detailed antisaccade parameters point to potentially underlying mechanisms, such as early pre-stimulus inhibition of the superior colliculus.

## Introduction

Obsessive-compulsive disorder (OCD) is a debilitating and often chronic psychiatric disorder characterized by obsessions (recurrent intrusive thoughts and/or images) and/or compulsions (ritualized repetitive behaviors), that affects 1–3% of the population worldwide (1). OCD is familial (2) with first-degree relatives having an approximately 5-fold increased risk of also being affected by the disease (3–5). Converging evidence from neuroimaging studies has suggested that altered functioning of the cortico-striato-thalamo-cortical (CSTC) circuits, including the orbitofrontal cortex (OFC), anterior cingulate cortex (ACC), basal ganglia and the thalamus, is implicated in the pathophysiology of OCD (6, 7). Specifically, an imbalance between the direct and indirect pathways within the CSTC circuits leads to an excess tone in the former over the latter, resulting in disturbances of executive functioning that may underlie features of the symptomatology of OCD (8). While the direct loop functions as a self-reinforcing positive feedback loop and contributes to the initiation and continuation of behaviors, the indirect loop serves as a mechanism of negative feedback, which is implicated in the inhibition of behaviors and in adaptive switching between behaviors (9, 10). The CSTC model of OCD thus predicts that affected individuals will be characterized by impaired performance in executive function tasks that demand the initiation of a volitional response while inhibiting a prepotent response.

A well-established approach to investigating those functions is the antisaccade task (11), which requires subjects to suppress a reflexive saccade toward a peripherally appearing stimulus in order to make a volitional eye movement in the opposite direction. As a control condition featuring the same setup but with no inhibitory demands, the prosaccade task has been used, which instructs the subject to look toward the appearing stimulus. While OCD patients perform normally with respect to prosaccade tasks in terms of error rates, latencies [for a review, see (12)] and intra-subject variability (ISV) of latencies (13), research employing the antisaccade task provides more mixed results, with a range of studies describing deficits in either error rates, latencies, both or neither (14–23). Inconsistencies in results may be explained by a variety of factors, including differences in task design parameters and sample characteristics. Across patients, obsessive-compulsive symptoms are highly heterogeneous, covering dimensions of washing/contamination, ordering/symmetry, forbidden thoughts and hoarding (24), which are all associated with differences in brain structure (25), brain function (26, 27) and neuropsychological performance (28). The interpretation of findings pertaining to the antisaccade task is further hindered by the tendency to utilize small sample sizes so that in certain cases, non-significant group differences might reflect poor statistical power rather than an absence of effect. Still, a quantitative meta-analysis of antisaccade performance in OCD is lacking, and the examination of larger samples of well-characterized OCD patients is warranted. The investigation of antisaccade performance in OCD could also benefit from examining more fine-grained outcome measures, like, for example, by subdividing direction errors into express errors and regular errors as distinct mechanisms appear to act as the foundation of these different types of errors (29). Errors with express latency (≤130 ms) result from failed preparatory suppression of the superior colliculus that must be present prior to stimulus appearance in order to prevent a reflexive orienting response toward the peripheral stimulus. Errors with regular latency (>130 ms), on the other hand, result from failure in active suppression of an automated saccade plan and the generation of a voluntary saccade to an abstract location. As these detailed saccade parameters have not been investigated in OCD patients so far, the analysis of express and regular error rates may point to specific neural mechanism deficient in OCD. Furthermore, functional and structural aberrations in OCD are not restricted to “executive” loops within the CSTC circuitry but also concern interconnected “affective” circuits that underlie functions of reward sensitivity, fear extinction and anxiety proneness (30, 31). Considering anxiety-related traits like harm avoidance in the context of antisaccade research may hence yield additional insights.

As response inhibition has been proposed as a potential endophenotype of OCD, the assessment of unaffected first-degree relatives might also prove fruitful in the search for the biological underpinnings of OCD. Endophenotypes are quantitative variables (e.g., cognitive or neurophysiological) associated with the disease while being distinct from the clinical phenotype itself (32). They are supposed to depend upon variation in fewer genes than the more complex disease phenotype and are therefore assumed to be more tractable to genetic analysis. Within families, endophenotypes and the disorder co-segregate, so that unaffected relatives are expected to show abnormalities similar to those observed in patients. In line with this concept, both OCD patients and relatives exhibit deficits in performance monitoring (33), planning capacity (34), cognitive flexibility (34–36) and response inhibition as evaluated with the Stop Signal Task (35, 37) and the Stroop Task (36). Most notably, Lennertz et al. (17) recently reported deficient antisaccade performance in OCD patients as well as in their unaffected first-degree relatives in terms of both elevated error rates and increased antisaccade latencies.

In the present study, we aimed to investigate whether patients with OCD and their first-degree relatives exhibit deficits in the antisaccade task by assessing error rates, mean latencies and ISV of latencies in a large and well-characterized sample. In addition, we conducted a quantitative meta-analysis to integrate our data with previous findings and quantify random and systematic influences on the results.

## Materials and methods

### Empirical study

#### Participants

One hundred and sixty-nine patients with OCD, 183 healthy comparison subjects and 100 unaffected first-degree relatives of OCD patients (*n* = 67 parents; *n* = 25 siblings; *n* = 8 offspring) participated in the study. Patients and controls were matched for age, gender and education. Relatives were significantly older than patients and controls (see Table 1 for sample characteristics). OCD patients and relatives were recruited via the outpatient clinics at the Department of Psychology of Humboldt-Universität zu Berlin and at the Department of Psychiatry and Psychotherapy of the University of Bonn, Germany. Healthy volunteers were recruited from the general population via public advertisements. A total of 279 subjects were assessed in Berlin (*n* = 101 OCD patients; *n* = 54 unaffected relatives; *n* = 124 control subjects) and 171 subjects were assessed in Bonn (*n* = 68 OCD patients; *n* = 46 unaffected relatives; *n* = 59 control subjects). All participants were examined by trained clinical psychologists using the Structured Clinical Interview for DSM-IV [SCID-I; (38, 39)]. To establish cross-site reliability of clinical ratings, all instructions were standardized and raters completed assessments of four training videos. Patients and relatives were only included if they were: (a) free of past or present psychotic, bipolar or substance-related disorders; (b) did not take neuroleptic medication for the previous 4 weeks; and (c) did not use benzodiazepines in the prior 2 weeks. Additionally, healthy controls were excluded if they: (a) took any psychoactive medication in the previous 3 months; (b) had a current Axis I disorder; (c) had a lifetime diagnosis of OCD or tic disorder; or (d) had a family history of OCD. All relatives were free of past or present OCD.

**Table 1:** Demographic and clinical characteristics of patients with OCD, unaffected first-degree relatives and healthy control subjects.

	**Patients with OCD**	**Unaffected first-degree relatives**	**Healthy control subjects**	**Statistic**	***p***
*N*	169	100	183		
Mean age, years (*SD*)	32.69 (10.44) [18–64]	46.55 (13.73) [18–67]	34.20 (12.70) [18–64]	*F*(2, 449) = 45.72	<0.001
Gender, % male	43.2	31.0	37.7	X^2^(2) = 4.00	0.14
Education (*SD*)^a^	4.90 (1.81) [1–7]	4.78 (2.00) [1–7]	5.17 (1.60) [1–7]	*F*(2, 447) = 1.88	0.15
Mean OCI-R score (*SD*)	27.76 (12.22) [5–64]	7.11 (6.76) [0–35]	4.57 (4.51) [0–22]	*F*_(2, 449)_ = 357.15	<0.001
Mean BDI-II score (*SD*)	18.46 (10.60) [0–45]	5.88 (6.96) [0–28]	2.96 (3.64) [0–18]	*F*_(2, 449)_ = 195.59	<0.001
Mean state anxiety score (*SD*)	42.32 (9.69) [22–72]	33.80 (7.52) [22–60]	31.39 (5.97) [20–52]	*F*_(2, 448)_ = 89.35	<0.001
Mean harm avoidance score (*SD*)	22.34 (6.65) [3–35]	14.61 (6.51) [1–31]	10.77 (5.22) [0–25]	*F*_(2, 445)_ = 160.33	<0.001
Mean MADRS score (*SD*)^b^	11.60 (8.60) [0–41]				
Mean Y-BOCS score (*SD*)^b^	22.03 (6.71) [0–35]^c^				
Mean age of onset (*SD*)^b^	20.93 (11.01) [3–59]				

*The range of scores is indicated in brackets. BDI-II, Beck Depression Inventory-II; MADRS, Montgomery Asberg Depression Rating Scale; OCD, obsessive-compulsive disorder; OCI-R, Obsessive-Compulsive Inventory-Revised; SD, standard deviation; Y-BOCS, Yale-Brown Obsessive Compulsive Scale*.

a *Education was assessed on a scale from 1 to 7*.

b *MADRS, Y-BOCS and age of onset were only assessed in patients*.

c *One patient had severe OCD in the past, but was fully remitted at the time of testing*.

Seventy-six OCD patients were medicated, with *n* = 62 taking selective serotonin reuptake inhibitors (SSRIs) and *n* = 33 receiving other antidepressants over the previous 4 weeks. Sixty-five patients were medication-naïve. Furthermore, the majority of patients had one or more comorbid Axis I disorders, with major depression being the most common comorbidity (*n* = 37 current episode; *n* = 66 remitted). Other current comorbidities included: panic disorder (*n* = 3 with agoraphobia; *n* = 5 without agoraphobia), social phobia (*n* = 13), specific phobia (*n* = 12), generalized anxiety disorder (*n* = 7), posttraumatic stress disorder (*n* = 6), attention deficit/hyperactivity disorder (*n* = 3), anorexia nervosa (*n* = 1), binge eating disorder (*n* = 2), tic disorder (*n* = 13), skin picking disorder (*n* = 10), hypochondria (*n* = 5), body dysmorphic disorder (*n* = 2), hoarding disorder (*n* = 4), pain disorder (*n* = 1), and unspecified somatoform disorder (*n* = 6).

The severity of OCD symptoms was evaluated with the German versions of the Yale-Brown Obsessive-Compulsive Scale [Y-BOCS; (40, 41)] and the Obsessive-Compulsive Inventory-Revised [OCI-R; (42, 43)]. Symptom dimensions were measured via the Y-BOCS Symptom Checklist [Y-BOCS CL; (40)]. The Montgomery Asberg Depression Rating Scale [MADRS;(44, 45)] and the Beck Depression Inventory-II [BDI-II; (46, 47)] were employed to assess the severity of current depressive symptoms. To account for potential effects of state anxiety, the State-Trait Anxiety Inventory [STAI; (48, 49)] was administered. Harm avoidance was assessed using the German version of the Temperament and Character Inventory [TCI; (50, 51)]. All participants had normal or corrected-to-normal vision and were free of any neurological disease (lifetime).

Written informed consent was obtained and participants were compensated for their time. The study was in accordance with the revised Declaration of Helsinki and approved by the local ethics committees of the Charité Universitätsmedizin Berlin and the University Clinic Bonn.

#### Eye movement recordings

Testing took place in a quiet, dimly lit room. Participants were seated comfortably in front of a 22-inch liquid crystal display (LCD) monitor (Viewsonic; height: 29.5 cm; width: 47.5 cm; resolution: 1,680 × 1,050 pixels; 60 Hz refresh rate) with a distance from eyes to screen of 70 cm. A chin rest was used to minimize head movements. At the Bonn assessment site, movements of the right eye were recorded using the EyeLink 1,000 system (SR Research, Mississauga, Ontario, Canada) at a sampling frequency of 1,000 Hz, whereas in Berlin, eye movements were recorded using the EyeLink II system (SR Research, Mississauga, Ontario, Canada) at a sampling rate of 250 Hz. Before the task started, the eye-tracker was calibrated with a five-point calibration task (0°, horizontal ± 13.3°, vertical ± 9.3°).

#### Saccade tasks

The saccade task was programmed with SR Research's Experiment Builder (version 1.10.1241). Pro- and antisaccades were evaluated via a blocked task design, with each block comprising 60 trials. At the beginning of each trial, participants were required to look at a central fixation cue (width and height: 0.46°), which remained on screen throughout each trial (overlap paradigm). After a random interval of 1,000–2,000 ms, a target stimulus (width and height: 0.46°) appeared in either the left or the right periphery at an angle of 16° and remained there for 800 ms. In the prosaccade block, subjects were instructed to make a saccade toward the peripheral target as fast and accurately as possible, whereas in the antisaccade block, subjects were required to make a saccade in the direction opposite of the target. Five practice trials were presented before each block in which the experimenter ensured that the subject had understood the instructions correctly. All stimuli were presented on a black background. Pro- and antisaccade blocks appeared in a fixed order as the strong main effect of task (latency of antisaccades > prosaccades) is well-established throughout the literature (52). Tasks were part of a larger oculomotor battery with additional tasks, of which the results were already (53, 54) and will be reported elsewhere.

#### Eye movement analysis

Saccades were identified according to SR Research's saccade detection algorithm (Data Viewer, version 1.11.900) and individually verified by a rater. Criteria for the identification of saccades were a velocity > 30°/s, an acceleration > 8,000°/s^2^, a minimum amplitude of 1° and a minimum latency to the peripheral stimulus of 80 ms (55). Trials including oculomotor events, i.e., saccades or blinks, <100 ms before stimulus onset were also excluded to ensure that subjects did not miss the onset of stimulus presentation [e.g., (56); on average 3.5% of trials were excluded in OCD patients, 4.6% in relatives and 2.8% in controls due to anticipatory eye movements]. Furthermore, trials were discarded if the gaze position at saccade onset deviated more than 2.3° (100 pixels) from the fixation stimulus position. Six subjects, who performed <15 valid trials for either of the tasks, were excluded from the analyses (57). For each subject, mean latencies and ISV of correctly performed pro- and antisaccades were calculated. Error rates were square-root transformed in order to obtain normal distribution and further subdivided into errors with express latency (≤130 ms) and regular latency [>130 ms; (55, 58)].

#### Statistical analyses

Statistical analyses were conducted using the Statistical Package for the Social Sciences (SPSS) Release 23.0 (SPSS Inc., Chicago, IL, USA). To test the hypothesis of impaired antisaccade performance in OCD patients, we conducted 2 × 2 analyses of variance (ANOVA) with group as the between-subjects factor (OCD patients vs. healthy comparison subjects) and task condition (prosaccades vs. antisaccades) as the within-subjects factor. Two distinct ANOVAs were carried out with mean saccade latency and ISV, i.e., *SD*, of saccades as dependent variables. In order to disentangle the interaction effects, *post-hoc t*-tests were conducted. Group differences in error rates were investigated with one-way ANOVAs. Results from these analyses were included in the combined meta-analysis, as described in section Statistical Methods.

Antisaccade performance of unaffected relatives was assessed via similar analyses. As relatives were significantly older than OCD patients and healthy volunteers, and age had a strong impact on saccade performance, it was included as a covariate for all ANOVAs that comprised relatives. To further support these results, additional analyses were conducted comparing unaffected relatives and a subsample of age-matched control subjects (*n* = 96).

Associations with specific OCD symptom dimensions were investigated using the Y-BOCS CL. Following the procedure established in previous studies (24, 59), the 13 main categories of the Y-BOCS CL were coded as 1 if the patient reported having experienced at least one symptom of the respective category. Otherwise, the category was coded as 0. These binary variables were then fed into an exploratory principal component analysis (PCA) with varimax rotation. Factors were extracted based on the Kaiser-Guttmann criterion, i.e., eigenvalue > 1. For each OCD patient, factor scores were exported for utility in further analyses.

Pearson's correlation coefficients were computed in order to explore relationships between mean saccade latencies and ISV, antisaccade error rates and continuous clinical variables. Group differences in demographic and clinical characteristics were tested using ANOVAs and Fisher's chi-square test was utilized to compare sex ratios across groups. We also performed exploratory ANOVAs using medication [medication-naïve (*n* = 65) vs. any psychoactive medication within the past 4 weeks (*n* = 81) vs. previous psychoactive medication but not within the past 4 weeks (*n* = 23)] and depressive comorbidity [current episode of major depression (*n* = 37) vs. remitted major depression (*n* = 66) vs. no lifetime diagnosis of major depression (*n* = 64)] as the between-subjects factor in OCD patients. Moreover, analyses were rerun excluding all OCD patients with current comorbid anxiety disorders, i.e., specific phobia, social phobia, panic disorder with/without agoraphobia and generalized anxiety disorder (*n* = 35). To investigate whether significant group differences in antisaccade performance were driven by increased levels of anxiety, correlations with state anxiety and harm avoidance were computed. The alpha level was set to 0.05 for all primary statistical analyses. In the correlation analysis evaluating OCD symptom dimensions, Bonferroni correction was applied to account for multiple comparisons.

### Meta-analysis

#### Study selection for meta-analysis

**Figure 1:**
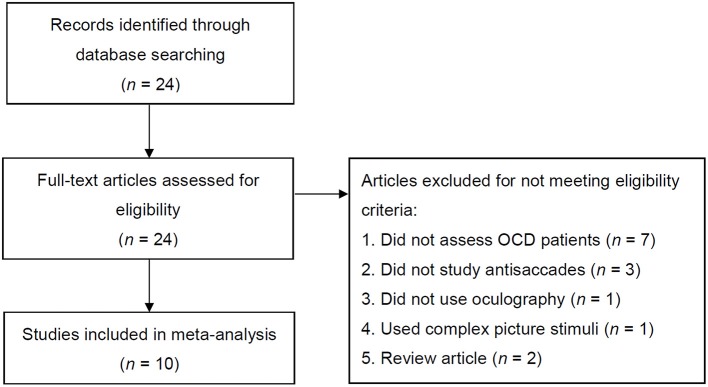
Flowchart showing the process of article selection. OCD, obsessive-compulsive disorder.

We conducted a systematic, comprehensive literature search of the PubMed database for relevant, full-length articles published up to the 30th April 2017 with the following search expressions: (“obsessive-compulsive disorder” OR “OCD” OR “obsessive-compulsive”) AND (“antisaccade” OR “anti-saccade” OR “saccade” OR “saccadic eye movement”). Publication reference lists of identified articles were searched as well. Only original research articles written in English and published in peer-reviewed journals were considered. The included studies were strictly those which made use of either video-oculography or electrooculography (EOG) to assess antisaccades. Studies employing complex picture stimuli as peripheral targets instead of simple cues were excluded. All studies were required to feature one group of individuals classified as having OCD based on a standardized clinical interview and one group of healthy (i.e., screened for the absence of a psychiatric or neurologic diseases) control subjects. Studies examining patient populations overlapping with previous publications were excluded. A flowchart showing the procedure of study selection according to the aforementioned criteria and PRISMA guidelines (60) is presented in Figure 1.

#### Data extraction

From each study, means and *SD*s or results from statistical tests (*t, F*, and *p* statistics) were extracted to compute the standardized mean difference (Cohen's *d*) in antisaccade latencies and error rates. As only one study reported ISV of antisaccade latencies and no study has investigated express and regular errors thus far, these measures could not be considered for meta-analysis. Additionally, we extracted the year of publication of each study, samples size, sex ratio as well as means and *SD*s of age and verbal intelligence quotient (IQ; or measures of education). If available, information on symptom severity as evaluated by Y-BOCS, the proportion of patients receiving psychotropic medication, the proportion of patients with comorbid depressive disorders, depressive symptom severity (e.g., according to the Hamilton Depression Rating Scale; HAM-D), age of OCD onset and task characteristics were extracted, too. The variables recorded were cross-verified by two researchers.

One study did not describe the main effect of group on antisaccade error rates but only the group × target amplitude interaction (20). As it was not possible to derive mean values and *SD*s across all three target amplitude conditions (8, 16, and 24°) from the information given, only results from the 16° condition were included in the meta-analysis. Similarly, McDowell and Clementz (19) reported statistics separately for each fixation cue condition (step, gap, overlap). For the sake of comparability with the present study's findings, data from the overlap condition were included in the meta-analysis. Furthermore, Agam et al. (14) reported antisaccade data from the same sample once acquired during electroencephalography (EEG) and once during functional magnetic resonance imaging (fMRI). As the EEG session was performed first, included more antisaccade trials and was presumably more comparable to the setup of the other studies, antisaccade data acquired during EEG were part of the present meta-analysis. Data from Maruff et al. (18) were read off figures as precisely as possible because the exact values could not be retrieved. Finally, two studies used square-root (17) and logit transformations (14), respectively, in order to obtain normal distributions of error rates. In these cases, effect sizes derived from analyses of the transformed values were featured in the meta-analysis, instead of raw values.

#### Statistical methods

Meta-analyses were conducted using the R-based software OpenMetaAnalyst (61). Effect sizes of outcome variables were estimated by computing the standardized mean difference (*SMD*), which is a measure of effect size calculated by subtracting the control group's mean from the OCD group's mean and dividing by the pooled *SD*. For each outcome measure within a study, i.e., antisaccade error rates and latencies, separate effect sizes were computed. Positive effect sizes indicated worse oculomotor functioning, i.e., higher error rates or longer latencies, in the OCD group relative to the control group, whereas negative effect sizes suggested better performance in OCD patients. With few exceptions, effect sizes were calculated directly from means and *SD*s reported in studies. When this information was not available, results of other statistical tests (e.g., *t* and *F* statistics) were consulted to calculate effect size (62). A weighted-average effect size was then computed for each outcome variable using the Hedges-Olkin random-effects method (63). To determine whether effect sizes were consistent across studies, we calculated the homogeneity statistics, *Q* and *I*^2^, where *I*^2^ = [(*Q*-df)/*Q*] × 100%, which describes the proportion of the variability in effect estimates that emanated from heterogeneity rather than sampling error.

Moreover, several meta-regressions were performed with mean age, proportion of males, proportion of medicated patients, mean Y-BOCS scores, task design (gap, step, overlap), number of trials, target amplitude and mean cue latency as moderators in order to investigate systematic influences of sample and task characteristics on antisaccade error rates and latencies.

## Results

### Empirical study

#### Effects of task, age and study site

Mean saccade latencies were significantly faster for the prosaccade task than the antisaccade task [*F*_(1, 451)_ = 1426.64, *p* < 0.001, η^2^ = 0.76, 95% CI (0.73, 0.79)]. A main effect of age indicated that saccade latencies rose with age [*F*_(1, 450)_ = 58.65, *p* < 0.001, η^2^ = 0.12, 95% CI (0.07, 0.17)]. Likewise, analysis of ISV of saccade latencies revealed a main effect of task type [*F*_(1, 451)_ = 135.08, *p* < 0.001, η^2^ = 0.23, 95% CI (0.17, 0.29)] with smaller ISV for the prosaccade task than the antisaccade task. ISV of saccade latencies significantly increased with age, as well [*F*_(1, 450)_ = 25.59, *p* < 0.001, η^2^ = 0.05, 95% CI (0.02, 0.10)]. Importantly, there was no effect of study site on any of the examined saccade variables (all *p* > 0.05).

#### Saccade performance in OCD patients and healthy comparison subjects

**Figure 2:**
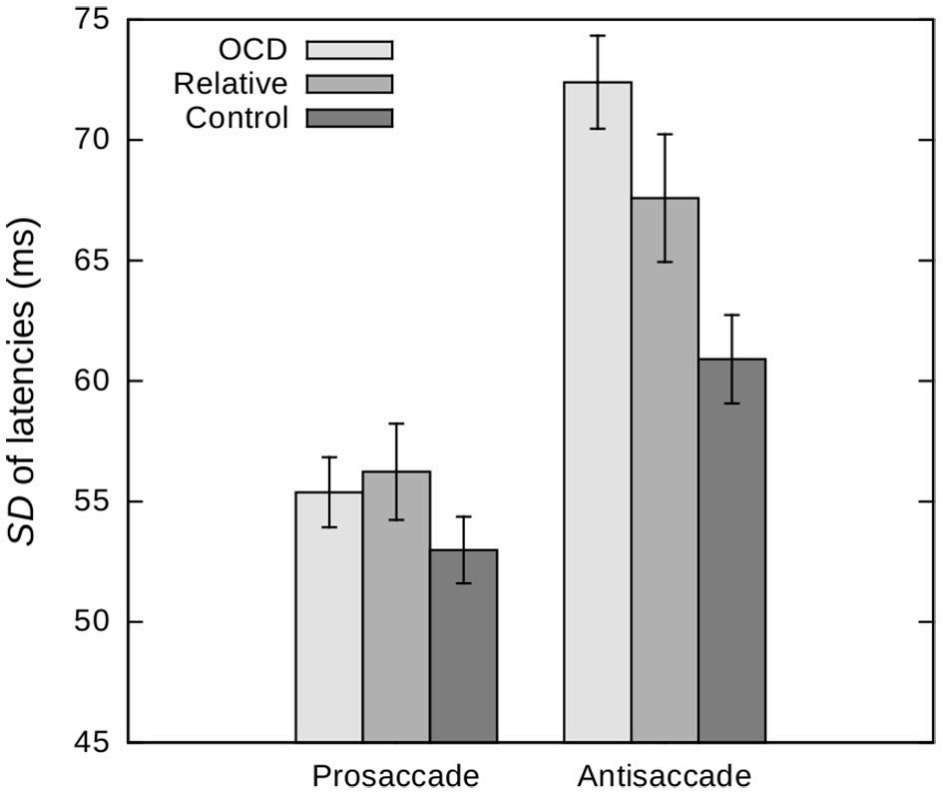
Intra-subject variability, i.e., *SD*s, of pro- and antisaccade latencies in patients with obsessive-compulsive disorder (OCD), unaffected first-degree relatives and healthy control subjects. Age is included as a covariate and values are depicted for a mean age of 36.37 years. Error bars indicate standard errors. The effect of group and the group by task interaction are significant at *p* = 0.002 and *p* = 0.001, respectively.

**Figure 3:**
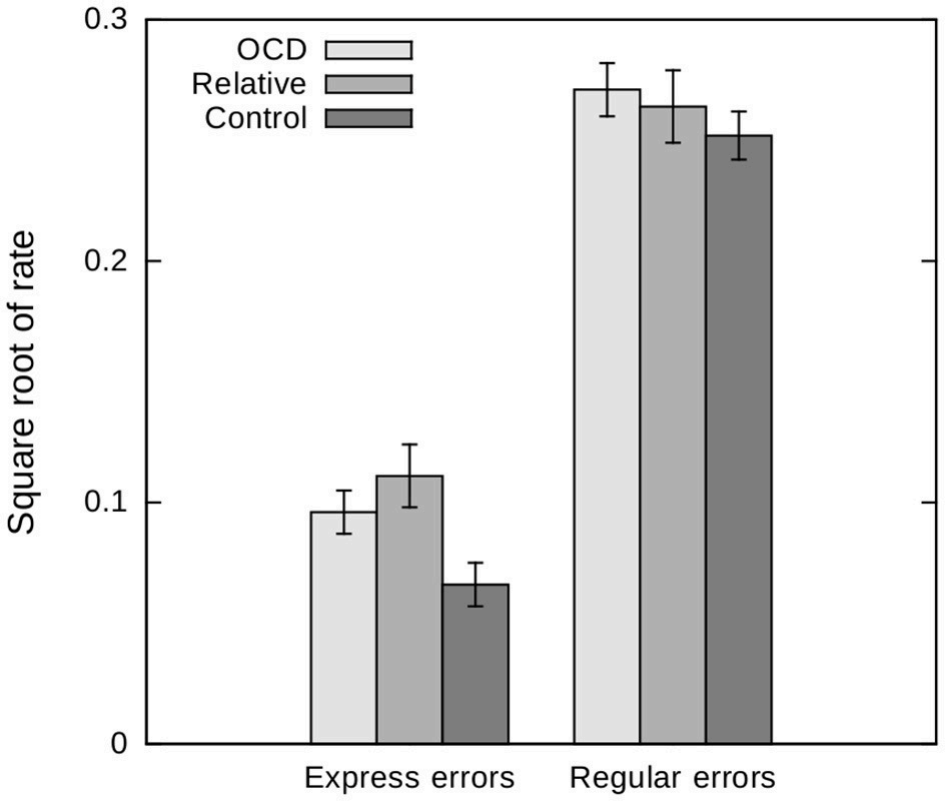
Express and regular error rates in the antisaccade task in patients with obsessive-compulsive disorder (OCD), unaffected first-degree relatives and healthy control subjects. Age is included as a covariate and values are depicted for a mean age of 36.37 years. Error bars indicate standard errors. Groups differ significantly regarding express errors (*p* = 0.007), but not regarding regular errors (*p* = 0.45).

Comparing mean saccade latencies between OCD patients and healthy controls yielded a significant group-by-task interaction [*F*_(1, 350)_ = 10.76, *p* = 0.001, η^2^ = 0.03, 95% CI (0.005, 0.07)]. In particular, patients had longer antisaccade latencies [*t*_(350)_ = 2.05, *p* = 0.042, *d* = 0.22, 95% CI (0.01, 0.43)], while there was no difference in prosaccade latencies between patients and controls [*t*_(329.08)_ = −1.32, *p* = 0.19, *d* = 0.14, 95% CI (−0.07, 0.01)]. Analysis of ISV yielded a significant effect of group [*F*_(1, 350)_ = 10.42, *p* = 0.001, η^2^ = 0.03, 95% CI (0.004, 0.07)] along with a significant group-by-task interaction [*F*_(1, 350)_ = 14.74, *p* < 0.001, η^2^ = 0.04, 95% CI (0.01, 0.09)] indicative of OCD patients exhibiting higher ISV in the antisaccade task compared to healthy controls [*t*_(315.39)_ = 4.06, *p* < 0.001, *d* = 0.44, 95% CI (0.23, 0.65); Figure 2], while ISV of prosaccades did not differ between groups [*t*_(350)_ = 1.01, *p* = 0.31, *d* = 0.11, 95% CI (−0.10, 0.32)]. Furthermore, patients made significantly more errors in the antisaccade task than controls [*t*_(322.47)_ = 2.09, *p* = 0.037, *d* = 0.22, 95% CI (0.01, 0.43)]. This effect was driven by express errors [*t*_(309.94)_ = 2.49, *p* = 0.013, *d* = 0.27, 95% CI (0.06, 0.48)], as groups did not differ significantly regarding regular errors [*t*_(350)_ = 1.17, *p* = 0.24, *d* = 0.12, 95% CI (−0.08, 0.33); Figure 3].

#### Saccade performance in unaffected relatives

Unaffected relatives did not differ from control subjects with regards to pro- and antisaccade latencies (*p* > 0.05 for both the main effect of group and the group-by-task interaction). However, relatives exhibited significantly higher ISV of latencies across tasks [*F*_(1, 280)_ = 4.62, *p* = 0.033, η^2^ = 0.02, 95% CI (0.0, 0.06); Figure 2]. While the group difference in overall error rate did not reach significance [*F*_(1, 280)_ = 1.40, *p* = 0.24, η^2^ = 0.005, 95% CI (0.0, 0.03)], separate analyses of express and regular errors suggested a significant effect of group on the rate of express errors [*F*_(1, 280)_ = 7.71, *p* = 0.006, η^2^ = 0.03, 95% CI (0.002, 0.07)] but not on the rate of regular errors [*F*_(1, 280)_ = 0.29, *p* = 0.59, η^2^ = 0.001, 95% CI (0.0, 0.02); Figure 3]. Relatives did not significantly differ from OCD patients across all measures (all *p* > 0.05).

These results were supported by additional analyses comparing unaffected relatives to a subsample of age-matched controls (*n* = 96). Groups did not differ with regards to pro- and antisaccade latencies (*p* > 0.05 for both the main effect of group and the group-by-task interaction), but relatives exhibited significantly higher ISV of latencies across tasks [*F*_(1, 194)_ = 6.33, *p* = 0.013, η^2^ = 0.03, 95% CI (0.0, 0.09)]. Again, the group difference in overall error rate did not reach significance [*t*_(194)_ = 1.84, *p* = 0.07, *d* = 0.26, 95% CI (−0.02, 0.54)], while separate analyses of express and regular errors yielded a significant effect of group on the rate of express errors [*t*_(186.88)_ = 2.93, *p* = 0.004, *d* = 0.42, 95% CI (0.14, 0.70)] but not on the rate of regular errors [*t*_(194)_ = 1.32, *p* = 0.19, *d* = 0.19, 95% CI (−0.09, 0.47)].

#### Effects of medication, depressive comorbidity, and OCD severity

Analyses of OCD patients with current, previous and no history of major depression did not yield any significant effects (all *p* > 0.05). Moreover, patients taking psychoactive medication did not differ from previously and never-medicated patients regarding antisaccade latencies, ISV and error rates (all *p* > 0.05). There were no significant associations between symptom severity as assessed by Y-BOCS or any of the saccade performance measures, either (all *p* > 0.05).

#### Effects of anxiety

Excluding OCD patients with current comorbid anxiety disorders did not alter the results substantially. Furthermore, there were no significant correlations between state anxiety and antisaccade performance in either OCD patients, unaffected relatives, or healthy controls (all *p* > 0.05). While we did not observe any associations between harm avoidance and antisaccade parameters in OCD patients and healthy controls (all *p* > 0.05), there was a significant positive correlation between harm avoidance and antisaccade error rate (*r* = 0.23, *p* = 0.020) in relatives, which was particularly pronounced with regards to express errors (*r* = 0.30, *p* = 0.002).

#### Symptom dimensions

PCA of Y-BOCS CL items yielded five factors representing the dimensions of ordering/symmetry/counting, checking/intrusive thoughts (aggression, religion, body), washing/contamination, hoarding and repeating/sexual obsessions. After controlling for multiple comparisons, none of the correlations between symptom dimensions and antisaccade performance parameters reached significance.

### Meta-analysis

#### Meta-analysis of previous studies

After standardized study selection (Figure 1), 10 studies were included in the meta-analysis with a total of 189 OCD patients and 204 control subjects. Samples and task characteristics are presented in Tables 2, 3.

**Table 2:** Sample characteristics of the 10 studies included in the meta-analysis.

**Author (year)**	***n* of OCD group**	***n* of control group**	**Mean age (*SD*) of OCD group**	**Mean age (*SD*) of control group**	**% male in OCD group**	**% male in control group**	**Mean estimated verbal IQ (*SD*) of OCD group**	**Mean estimated verbal IQ (*SD*) of control group**	**Depressive comorbidity**	**Mean depressive symptom severity (*SD*)**	**Mean OCD symptom severity as assessed by Y-BOCS (*SD*)**	**Mean age of OCD onset (*SD*)**	**Medication**	**OCD symptom dimensions**
(22)	11	14	39 (7)	38 (10)	45.5 or 54.4 %^a^	42.9 or 50.0 %^a^	similar education		–	–	24.4 (4.5)	–	*n* = 1 none, *n* = 10 AD	–
(19)	12	12	32.7 (10.4)	37.0 (15.3)	58 %	42 %	–	–	–	HAM-D: median = 8	O: median = 11 C: median = 11	–	*n* = 4 none, *n* = 8 psychotropic medication	–
(20)	12	12	30.1 (9.4)	30.2 (9.0)	50.0 %	50.0 %	similar socioeconomic status		*n* = 1 dysthymia	HAM-D: median = 7	O: median = 11 C: median = 10	21.0 (9.2)	all medication-free	–
(18)	12	12	47.0 (8.5)	46.0 (9.9)	41.7 %	41.7 %	similar education		–	HAM-D: 9.0 (4.4)	24.2 (10.3)	20.7 (13.4)	*n* = 3 none, *n* = 9 AD	–
(21)	22	24	34.2 (11.2)	31.0 (6.3)	59.1 %	62.5 %	–	–	*n* = 1 MDD, *n* = 7 dysthymia	–	20.1 (7.7)	–	*n* = 2 none, *n* = 20 AD	–
(23)	14	14	29.1 (7.2)	28.4 (6.28)	64.3 %	64.3 %	–	–	all comorbidity-free	HAM-D: 8.0 (4.3)	23.7 (3.8)	*n* = 8 juvenile, *n* = 6 adult	all psychotropic-naïve	–
(16)	30	30	32.3 (9.8)	32.8 (9.2)	36.7 %	36.7 %	105.4 (9.6)	106.9 (8.8)	–	MADRS: 4.9 (6.2)	18.0 (7.0)	18.1	*n* = 15 none, *n* = 15 AD	mixed (OCI-R)
(17)	21	21	38.9 (6.9)	41.2 (13.0)	47.6 %	38.1 %	114.7 (12.8)	116.6 (12.4)	0 ≤ *n* ≤ 8 MDD^b^	BDI: 11.86 (8.71)	17.2 (8.3)	–	13 ≤ *n* ≤ 21 AD^b^	mixed (Y-BOCS Checklist)
(14)	21	20	33 (11)	33 (11)	38.1 %	55.0 %	110 (11)	113 (6)	*n* = 1 MDD, *n* = 4 dysthymia	BDI-II: 13 (9)	23 (5)	–	*n* = 14 none, *n* = 6 AD, *n* = 1 AD + memantine	*n* = 10 washing, *n* = 9 checking + obsessions, *n* = 2 symmetry
(15)	34	45	23.2 (3.4)	23.2 (1.3)	100.0 %	100.0 %	–	–	–	–	range = 14–33	–	*n* = 6 none, *n* = 16 AD, *n* = 7 AD + AP, *n* = 3 AD + MS, *n* = 1 AD + AP + MS, *n* = 1 buspirone	–

*AD, antidepressants; AP, antipsychotics; BDI, Beck Depression Inventory; C, compulsions subscale of the Y-BOCS; HAM-D, Hamilton Depression Rating Scale; MADRS, Montgomery Asberg Depression Rating Scale; MS, mood stabilizers; O, obsessions subscale of the Y-BOCS; OCD, obsessive-compulsive disorder; OCI-R, Obsessive-Compulsive Inventory-Revised; SD, standard deviation; Y-BOCS, Yale-Brown Obsessive Compulsive Scale*.

*Hyphens indicate that the specific characteristic was not reported*.

a *The information given in the main text and figures is divergent*.

b *Characteristics were only available for the full sample, but not for the subsample which completed the antisaccade task*.

**Table 3:** Task characteristics and results of the 10 studies included in the meta-analysis.

**Author (year)**	**Task design**	***n* of trials**	**Target amplitude**	**Cue latencies**	**Results: mean latencies**	**Results: error rates**	**Results: *SD* of latencies**
(22)	step	10–16	~10°	–	–	OCD > CON	–
(19)	step, gap, or 200 ms overlap	120	10, or 20°	2,000–2,500 ms	OCD > CON	OCD = CON	–
(20)	step	36	8, 16, or 24°	1,500–2,500 ms	OCD = CON	OCD > CON (8°) OCD = CON (16°) OCD = CON (24°)	–
(18)	step	106	10, or 15°	2,000–2,500 ms	OCD > CON	OCD = CON	–
(21)	step	20	12°	700, 1,000, or 1,300 ms	OCD = CON	OCD = CON	–
(23)	200 ms gap	50	7°	1,000 ms	OCD > CON	OCD = CON	–
(16)	overlap	40	–	1,000–2,000 ms	OCD = CON	OCD = CON	–
(17)	200 ms overlap	50	16°	1,500, 2,000, 2,500, or 3,000 ms	OCD > CON (only interaction term with prosaccades)	OCD > CON	–
(14)	200 ms gap; 50% trials with distractor; 40% without distractor, 10% fake-hard; twice: during fMRI and EEG	384 (EEG) 512 (fMRI)	10°	2,000 ms	OCD = CON (EEG) OCD = CON (fMRI)	OCD = CON (EEG) OCD > CON (fMRI)	–
(15)	step	90	2–10° (1° intervals)	1,000–2,000 ms	OCD = CON	OCD > CON	OCD > CON

*CON, healthy control subjects; OCD, obsessive-compulsive disorder; SD, standard deviation*.

*Hyphens indicate that the specific characteristic was not reported*.

Meta-analysis of antisaccade error rates yielded a weighted-average *SMD* of 0.55 [95% CI (0.28, 0.81), *p* < 0.001]. According to the *Q* and *I*^2^ statistics, results across the studies were moderately, though not significantly, heterogeneous [*Q*_(9)_ = 12.71, *I*^2^ = 35.32%, *p* = 0.18]. As indicated by the meta-regressions, there was no significant impact of age (*b* = 0.012, *p* = 0.55), gender (*b* = 0.002, *p* = 0.74), medication (*b* = 0.45, *p* = 0.24) and Y-BOCS score (*b* = 0.024, *p* = 0.67). In terms of task characteristics, there was no effect of task design (*p* = 0.95), number of trials (*b* = 0.001, *p* = 0.60), target amplitude (*b* = −0.015, *p* = 0.68) and mean cue latency (*b* = 0.000, *p* = 0.095).

Meta-analysis of antisaccade latencies yielded a similar *SMD* of 0.56, but the effect did not reach significance [95% CI (−0.06, 1.17), *p* = 0.075] as considerable statistical heterogeneity was observed [*Q*_(8)_ = 36.12, *I*^2^ = 87.26%, *p* < 0.001]. Meta-regressions using mean age (*b* = 0.067, *p* = 0.078), proportion of males (*b* = −0.006, *p* = 0.67), proportion of medicated patients (*b* = −0.17, *p* = 0.83) and mean Y-BOCS scores (*b* = 0.16, *p* = 0.20) as moderators did not yield any significant effects on *SMD*. Furthermore, there was no effect of task design (*p* = 0.95), number of trials (*b* = −0.001, *p* = 0.80), target amplitude (*b* = 0.006, *p* = 0.94) and mean cue latency (*b* = 0.001, *p* = 0.16).

#### Meta-analysis of previous and the present empirical study

**Figure 4:**
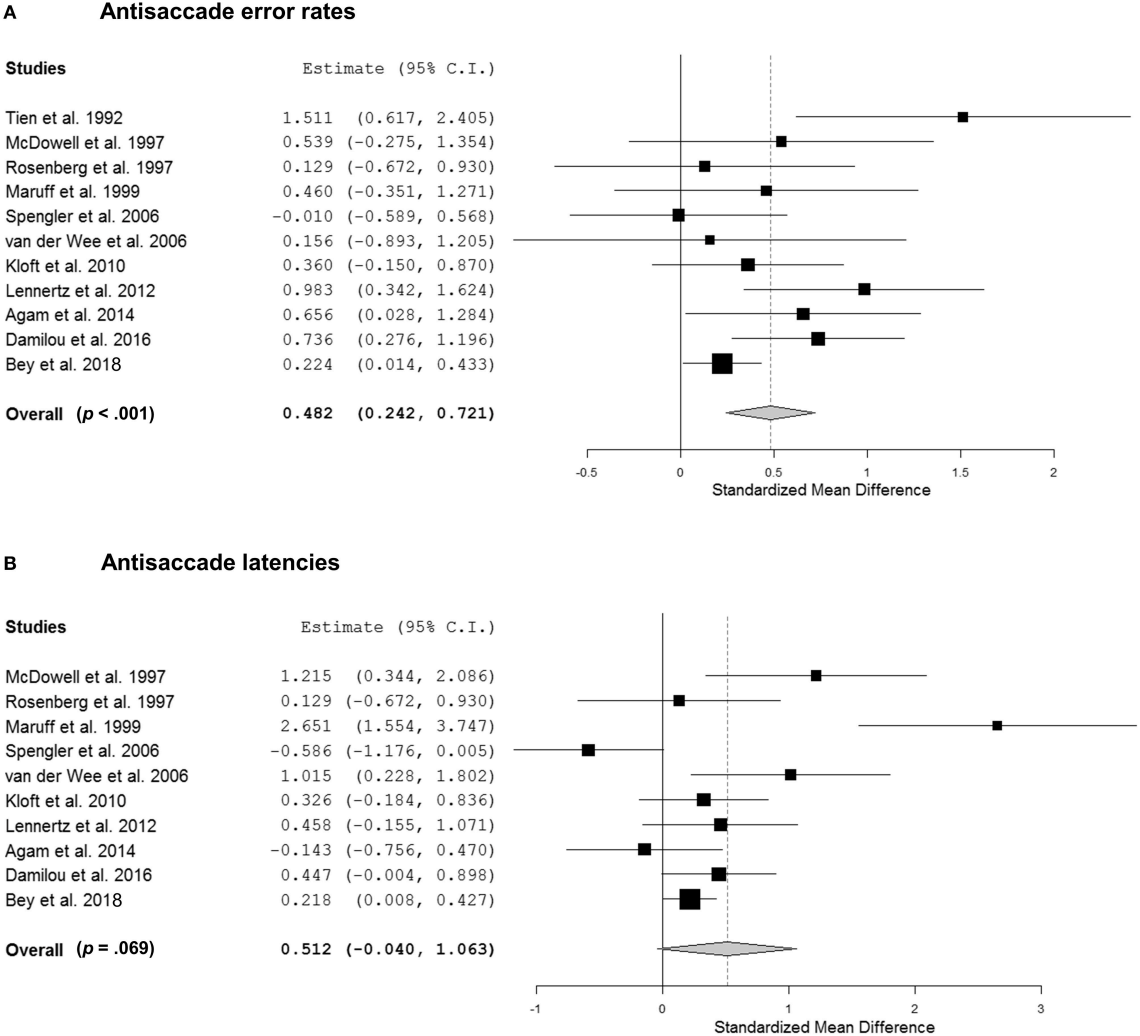
**(A)** Forest plot showing effect sizes for antisaccade error rates in patients with obsessive-compulsive disorder (OCD) as compared to healthy control subjects. Higher values indicate higher error rates in patients. **(B)** Forest plot portraying the effect sizes for antisaccade latencies in patients with OCD as compared to healthy control subjects. Higher values indicate longer latencies in patients.

Including the present empirical study in the meta-analysis of antisaccade error rates yielded a weighted-average *SMD* of 0.48 [95% CI (0.24, 0.72), *p* < 0.001; Figure 4A]. Again, we noted moderate, though non-significant, heterogeneity across studies [*Q*_(9)_ = 17.22, *I*^2^ = 45.03%, *p* = 0.070]. As indicated by the meta-regressions, there was no significant effect of age (*b* = 0.012, *p* = 0.56), gender (*b* = 0.005, *p* = 0.34), medication (*b* = 0.55, *p* = 0.15) and Y-BOCS score (*b* = 0.010, *p* = 0.86). While there was no impact of task design (*p* = 0.84), number of trials (*b* = 0.001, *p* = 0.48) and target amplitude (*b* = −0.04, *p* = 0.050) on *SMD*, the effect of mean cue latency (*b* = 0.000, *p* = 0.035) reached significance. Specifically, longer cue latencies were associated with greater *SMD*s.

Meta-analysis of antisaccade latencies yielded a weighted-average *SMD* of 0.51 [95% CI (−0.04, 1.06), *p* = 0.069; Figure 4B]. The *Q* and *I*^2^ statistics indicate that results across studies were significantly heterogeneous [*Q*_(9)_ = 37.14, *I*^2^ = 90.20%, *p* < 0.001]. Meta-regressions utilizing mean age (*b* = 0.065, *p* = 0.072), proportion of males (*b* = −0.003, *p* = 0.77), proportion of medicated patients (*b* = −0.18, *p* = 0.80) and mean Y-BOCS scores (*b* = 0.14, *p* = 0.20) as moderators did not yield any significant effects on *SMD*. With respect to task characteristics, there was no effect of task design (*p* = 0.98), number of trials (*b* = −0.000, *p* = 0.82), target amplitude (*b* = −0.008, *p* = 0.91) and mean cue latency (*b* = 0.001, *p* = 0.12).

## Discussion

The present study serves as an extensive investigation of antisaccade performance in OCD patients and their unaffected first-degree relatives. Convergent findings from meta-analysis and a large empirical study indicate that patients with OCD exhibit significantly increased error rates in the antisaccade task while response latencies are not consistently elongated. Whereas effect sizes for error rates and mean latencies appear to be just moderate, we observed pronounced group differences with regards to ISV of antisaccade latencies in OCD patients as well as in unaffected relatives, providing the first evidence for a potential endophenotype of OCD. Furthermore, detailed analyses of error rates indicate that both patients and first-degree relatives exhibit more express errors in the antisaccade task than healthy controls.

Our findings highlight that antisaccade deficits in OCD are substantial, but only of moderate effect size and limited by inconsistencies across studies. The meta-analysis of 10 studies showed that patients with OCD feature a significantly increased antisaccade error rate compared to matched healthy controls. With regards to antisaccade latencies, however, the group difference did not reach significance as there was large heterogeneity across studies. As indicated by meta-regression, age, gender, medication status and OCD severity did not contribute to the variability in effect sizes, suggesting that OCD patients' deficits in the antisaccade task are not moderated by demographic variables. This observation is in agreement with the meta-analyses of cognitive function in OCD (64, 65) and the absence of associations between patients' medication status, depressive comorbidity, OCD severity and oculomotor performance in our empirical data. Still, other clinical variables which are less state-dependent and more strongly related to the genetic underpinnings of OCD, such as age of onset (66) and symptom dimensions (67), could not be analyzed via meta-regressions based on the small number of studies reporting relevant information. With respect to our empirical investigation, however, we did not observe any significant correlations between OCD symptom dimensions and antisaccade performance after controlling for multiple comparisons.

While the majority of task characteristics did not affect *SMD* as was indicated by the meta-regressions, the effect of mean cue latency reached significance when the present empirical study was included. Accordingly, longer mean cue latencies were associated with greater group differences in error rates. Though longer cue latencies are not generally associated with higher error rates or an increased frequency of express saccades, OCD patients and controls might be differentially affected by the manipulation of cue latencies, resulting in a growing group difference.

Our empirical data indicate that the increased antisaccade error rates in OCD patients are primarily driven by express latency errors. As reviewed by Coe and Munoz (29), express and regular errors arise from failures of different forms of suppression mechanisms, which can be investigated via neurophysiological experiments in macaques. At the start of an antisaccade trial, pre-emptive top-down inhibition of saccade-generating neurons in the frontal eye fields and the intermediate layers of the superior colliculus (SCi) must be present before the stimulus appears in order to prevent express errors. After the stimulus' appearance, voluntary antisaccade commands must compete with, and override, automated visually initiated saccade commands to prevent longer latency errors. Potential sources of pre-stimulus inhibition comprise fixation neurons within the SCi, which are tonically active during visual fixation and cease firing during the execution of saccades (68). Hence, the inhibition of neurons in the SCi prior to stimulus elicitation is crucial to preventing the initial visual transient response from triggering a direction error (29). The CSTC model of OCD is in accordance with the observed excess in express errors. Specifically, imbalances between the direct and indirect CSTC pathways may not only result in a reduced inhibition of the thalamus, but may also contribute to a diminished inhibition of the SCi during the antisaccade task, which may hence cease firing more easily and facilitate the execution of quick, reflexive saccades toward the target. Notably, oculomotor regions of the frontal cortex and the basal ganglia also feature pre-stimulus activity, establishing a form of top-down inhibition that appears to be required for the inhibition of the SCi (29). A deficient transmission of inhibitory signals from frontal regions to the SCi via the CSTC circuitry may hence foster an increased frequency of express error rates. Evidence from neuroimaging indicates that both OCD patients and their unaffected relatives show aberrations in CSTC functional connectivity (69), which may serve as the basis for the increased express error rates we observed. In conclusion, our findings support the role of the antisaccade error rate as a potential endophenotype of OCD (17) and point to a more particular mechanism, i.e., early pre-stimulus inhibition of the SCi.

Aberrant CSTC function may also contribute to the increased ISV of correct antisaccade latencies. To our knowledge, this is the first study to demonstrate that ISV in antisaccade latencies constitutes a potential endophenotype of OCD. While increased *SD*s of antisaccade latencies have previously been described in OCD patients (15), unaffected first-degree relatives have not been evaluated thus far. In general, ISV in reaction times is most strongly observed in tasks that require executive control (70), indicating lapses of attention or cognitive control (71, 72). ISV of response times is sensitive to frontal dysfunction (73), increased in patients with schizophrenia (15) and also linked with the genetic risk of schizophrenia (74). Considering the strong genetic overlap between schizophrenia and OCD (75), endophenotypes of schizophrenia could prove informative for OCD, as well. In fact, endophenotypes have the potential to provide measures that are sensitive to multiple diagnostic constructs, and may aid the identification of shared pathomechanisms (76). Trans-diagnostic approaches of neurocognitive endophenotypes have been proposed, entailing instructive implications for the future classification of psychiatric disorders, genetics and therapeutics (77).

Twin studies of OCD and OCD-related traits yield heritability estimates of roughly 50% (78, 79), but until now, the identification of the specific genetic variants underlying this heritability has been difficult. Two genome-wide association studies (GWAS) studies and a recent meta-analysis of them did not provide evidence for genome-wide significant hits (80–82), possibly because of still insufficient sample sizes (*n* = 2,688 OCD patients and *n* = 7,037 controls). In recent years, this issue has been addressed by an endeavor to identify endophenotypes as potential vulnerability factors of OCD, which are presumably less genetically complex and hence more tractable to genetic analysis. According to established criteria, an endophenotype must: (a) be heritable; (b) be associated with the illness; (c) be independent of clinical state; (d) co-segregate with the illness within a family; and (e) represent reproducible measurements (32, 76). Antisaccade performance, specifically error rates, meets each of these criteria, rendering it a promising endophenotype of OCD: (a) with heritability estimates ranging from 42 to 61%, antisaccades have a strong genetic component (83–85); (b) antisaccade deficits have repeatedly been observed in patients with OCD; (c) OCD symptom severity is not correlated with antisaccade performance; (d) first-degree relatives of OCD patients exhibit worse performance than healthy subjects from the general population [(17); present study]; and (e) antisaccade error rates and the *SD*s of antisaccade latencies are sufficiently reliable (57, 86). Until now, antisaccade error rates are the most commonly researched outcome measure in the antisaccade task, while ISV of antisaccade latencies and express error rates have rarely been examined in endophenotype studies. Our findings highlight that OCD patients as well as their first-degree relatives exhibit prolonged *SD*s of antisaccade latencies and increased express error rates, independent of symptom severity. Further research investigating the genetic basis of these more detailed outcome measures is warranted. The genetic architecture of OCD appears to be highly polygenetic and primarily constituted of common variants 87, 88, with a SNP heritability that is among the highest of all psychiatric disorders (89). Future studies may look for associations between polygenic risk scores of OCD and antisaccade performance so as to assess shared genetic contributions. The identification of risk genes will contribute to the understanding of etiological mechanisms in OCD and may eventually point to new targets for medication.

In order to explore the potentially mediating role of anxiety, we assessed correlations between antisaccade performance, state anxiety, and harm avoidance, a personality trait that has previously been discussed as an endophenotype of OCD [(90) sample overlapping with the present sample; (91)]. While we did not observe any associations between state anxiety and antisaccade parameters within groups, there was a significant positive correlation between harm avoidance and antisaccade error rate in relatives, which was particularly pronounced with regards to express errors. Hence, the underperformances we observed in relatives of OCD patients appear to be driven by a shared vulnerability expressed in an anxious personality rather than by state anxiety. In accordance with the assumption of pleiotropy, the genetic risk for OCD that is presumably reflected in elevated scores of harm avoidance may at the same time contribute to elevated antisaccade error rates.

Concerning antisaccade latencies, the findings are less consistent. Although we observed slightly increased antisaccade latencies in OCD patients within our empirical sample, meta-analysis did not demonstrate a significant effect stemming from large heterogeneity. While the majority of studies examined antisaccade latencies without considering prosaccade latencies, our results indicate that using a repeated-measures model, including the saccade task as a between-subjects factor, yields more pronounced group differences between OCD patients and controls. Whereas the mere comparison of antisaccade latencies barely reached significance, including prosaccade latencies in the model led to the observation of a strong group difference. Though groups did not differ significantly with respect to prosaccade latencies, the slightly faster prosaccades in patients contributed to the extensive group-by-saccade-task interaction effect. These results are in line with (17), who also observed a more pronounced effect when a repeated-measures approach was employed. Hence, disregarding prosaccades as a reference task may explain why the overall latency effect did not reach significance in the meta-analysis.

The present study is not without limitations. First, relatives were significantly older than patients and controls. In order to address this shortcoming, age was included as a covariate across all analyses comprising relatives, and additional analyses were conducted comparing relatives to an age-matched subsample of control subjects. Notably, patients and controls were well-matched with regards to age so the main analyses were not affected by this issue. Furthermore, the mean age of unaffected relatives lies well above the average age of onset of OCD symptoms, making it very unlikely that the effects are driven by subjects who will develop OCD later on. Second, a substantial number of patients was medicated with SSRI or other antidepressants. Though SSRI have been shown to influence functional brain networks (92), they do not seem to affect antisaccade performance (93). In line with this notion, we did not observe significant differences between medication-naïve, currently medicated and previously medicated OCD patients. Third, as many of the original studies appear to be underpowered, the power of the meta-analyses is likely also relatively low.

In summary, combining meta-analysis of previous findings with the assessment of a large and thoroughly characterized empirical sample allowed for an extensive examination of antisaccade performance in OCD while considering a large variety of covariates, including medication status, depressive comorbidity, comorbid anxiety disorders and OCD symptom dimensions. Our results indicate that antisaccade deficits in OCD are substantial, though of moderate effect size. Assessing more detailed parameters, such as express error rates and ISV of saccades, has shown to be informative, as we found first evidence that an increased ISV of antisaccade latencies and an elevated rate of express errors constitute potential endophenotypes of OCD.

## Data availability

The raw data supporting the conclusions of this manuscript will be made available by the authors, without undue reservation, to any qualified researcher.

## Author contributions

KB, LL, UE, NK, and MW contributed conception and design of the study; KB, LL, and IM programmed the saccade task; KB conducted the statistical analyses and wrote the first draft of the manuscript; KB, CK, LL, AR, JK, SH, and RG were involved in data-acquisition for this bi-centric study. All authors contributed to manuscript revision, read and approved the submitted version.

### Conflict of interest statement

The authors declare that the research was conducted in the absence of any commercial or financial relationships that could be construed as a potential conflict of interest.
